# Identification of *doublesex* alleles associated with the female-limited Batesian mimicry polymorphism in *Papilio memnon*

**DOI:** 10.1038/srep34782

**Published:** 2016-10-06

**Authors:** Shinya Komata, Chung-Ping Lin, Takuro Iijima, Haruhiko Fujiwara, Teiji Sota

**Affiliations:** 1 Department of Zoology, Graduate School of Science, Kyoto University, Kyoto, Japan; 2 Department of Life Science, National Taiwan Normal University, Taipei, Taiwan; 3 Department of Integrated Biosciences, University of Tokyo, Kashiwa, Japan

## Abstract

The female-limited Batesian mimicry polymorphism in *Papilio* butterflies is an intriguing system for investigating the mechanism of maintenance of genetic polymorphisms. In *Papilio polytes*, an autosomal region encompassing the sex-determinant gene *doublesex* controls female-limited mimicry polymorphism. In the closely related species *P. memnon*, which also exhibits female-limited Batesian mimicry polymorphism, we identified two allelic sequences of the *doublesex* gene that corresponded exactly with the mimetic and non-mimetic female phenotypes. Thus, the genetic basis of the mimicry polymorphism in *P. memnon* is similar to that in *P. polytes.* However, the mimetic and non-mimetic alleles of the two species were not identical, and the divergence of alleles occurred independently in *P. memnon* and *P. polytes*. Different mutation-selection processes may have resulted in the convergent patterns of mimicry polymorphism in these *Papilio* butterflies.

Batesian mimicry is the phenomenon in which palatable mimics avoid predation by resembling unpalatable species^1^,^2^,^3^. One of the most intriguing types of Batesian mimicry in butterflies is the female-limited polymorphism in which females display both mimetic and non-mimetic forms^4^,^5^,^6^. Some *Papilio* butterflies are textbook examples of female-limited Batesian mimicry polymorphism, which is controlled by a supergene locus^7^,^8^,^9^,^10^,^11^. Recently, the genetic basis of Batesian mimicry was revealed in two *Papilio* species, *P. polytes* and *P. dardanus*, and the allelic differences associated with mimicry polymorphism have been identified^12^,^13^,^14^. In *P. polytes*, a single ~130-kb autosomal region including the sex-determinant gene *doublesex (dsx*), with mimetic (*H*-type) and non-mimetic (*h*-type) allelic sequences, controls polymorphism, and these two alleles are protected against recombination by an inversion that covers the entire region of the *dsx* gene^12^,^14^. These findings would facilitate the comparative genomic study of female-limited mimicry polymorphisms in *Papilio* butterflies to understand the evolutionary diversification of genes underlying the complex adaptive traits involved in mimicry.

The Great Mormon butterfly, *Papilio memnon*, is closely related to *P. polytes* and exhibits similar female-limited Batesian mimicry polymorphism^15^,^16^. *P. memnon* females can exist as either the mimetic or non-mimetic form, whereas *P. memnon* males exhibit only the non-mimetic form (Fig. 1). Mimetic females have tails in their hindwings and aposematic colours in their hindwings and abdomens (Fig. 1), which allow them to mimic unpalatable species in the genera *Pachliopta* and *Atrophaneura*^9^,^11^. These phenotypic traits are tightly linked with a putative supergene^9^,^11^. Based on the phylogenetic proximity of *P. polytes* and *P. memnon* (Fig. 2), we predicted that the genetic basis of female-limited Batesian mimicry in *P. memnon* would be the same as that in *P. polytes* and that *P. memnon* would also have *doublesex* alleles corresponding to mimetic and non-mimetic forms.

In this study, we first sequenced mRNA from the wing discs of *P. memnon* pupae to identify *dsx* ortholog sequences. Next, we identified specific sequences in mimetic (*H*) and non-mimetic (*h*) alleles by sequencing cDNA derived from mRNA from reared individuals. Second, we sequenced a portion of *dsx* exon 1 using genomic DNA from field-collected individuals to confirm that the mimetic phenotype corresponded with the *dsx* allele type. Finally, we performed high-resolution melting (HRM) analysis to determine *dsx* genotypes from genomic DNA of males and females collected in the field, and investigated geographic variation in the frequency of the mimetic allele in *P. memnon* populations of Taiwan and Okinawa, Japan.

## Results

Our *de novo* assembly of 101-bp paired-end reads from *P. memnon* transcriptomes (DDBJ DRA accession nos.: SAMD00052594-SAMD00052595) yielded 45,887 and 39,035 contigs for one female and one male, respectively (Table S1, Supplementary Information). In a BLAST search of the *dsx* sequences, two of the female contigs (c1658_g1_i1, c17110_g5_i1) yielded significant matches with the F1 isoform of the *dsx H* and *h* alleles of *P. polytes*, but no male contig yielded a significant match (Table S2, Supplementary Information). The two female contigs were identified as portions of the *dsx* sequence separated by a 59-bp gap. Upon sequencing of a 567-bp portion of contig c1658_g1_i1, we obtained three heterozygous and three homozygous sequences from 6 female cDNA samples and six heterozygous and five homozygous sequences from 11 male cDNA samples. These sequences were comprised of only two genotypes, probably corresponding to *Hh* and *hh*. Next, we discriminated the *H* and *h (hh*) sequences by subtracting the *h* sequence from the *Hh* sequence (Fig. 3; DDBJ accession: LC155217-LC155218). The 567-bp sequence contained 18 nucleotide substitutions (3.2% of all bp) between *H* and *h*, of which 4 were non-synonymous (Fig. 3). In this region, the *P. memnon H* sequence differed from that of *P. polytes* by 57 nucleotide substitutions (10.1%), of which 13 were non-synonymous. The *P. memnon h* sequence differed from that of *P. polytes* by 33 nucleotide substitutions (5.8%), of which 5 were non-synonymous. None of the non-synonymous substitutions between the *H* and *h* sequences were shared between the two species. The *P. polytes h* sequence contained a 14-bp insertion in the 5′-UTR (untranslated region) that was absent in *P. memnon*. The reconstructed phylogenetic relationship among the four *dsx* allelic sequences from *P. memnon* and *P. polytes* revealed that the divergence of *H* and *h* occurred independently within each species (Fig. 4).

Sequencing of the 185-bp portions (containing 10 single-nucleotide polymorphisms) from 134 wild-caught individuals again yielded three sequence types, corresponding to the putative *HH, Hh* and *hh* genotypes. In females, the *HH* and *Hh* genotypes were obtained from the mimetic form and the *hh* genotype from the non-mimetic form (Table 1). Both the *H* and *h* alleles were found in Taiwan, but not in Okinawa, where no mimetic females occur (Table 1).

HRM analysis using primers targeting the *dsx* sequences of *P. memnon* showed that the *H* and *h* alleles exhibited unique, non-overlapping melting curves, enabling differentiation of the three *dsx* genotypes (Fig. S1, Supplementary Information). The sensitivities of the delta *T*_*m*_ values and shape discriminations were 40% and 20%, respectively. HRM analysis of the 134 wild-caught individuals afforded genotyping data consistent with those obtained by direct sequencing in all but one sample (Table 1). This inconsistent sample was genotyped as *Hh* by direct sequencing but as *HH* by HRM analysis.

In Taiwan, the proportions of *dsx* genotypes among males and females did not significantly differ from Hardy-Weinberg equilibrium at all localities (Fisher’s exact probability test; *P* > 0.05). The estimated frequency of the *H* allele ranged from 0.36 to 0.55 in males (Table 1). The *dsx* allele frequencies in males did not significantly differ among nine localities (Fisher’s exact probability test; *P* = 0.93). The estimations based on females were unreliable because of the small sample sizes; the total number of females collected in Taiwan was only one-third the number of males (Table 1).

## Discussion

**Figure 2:**
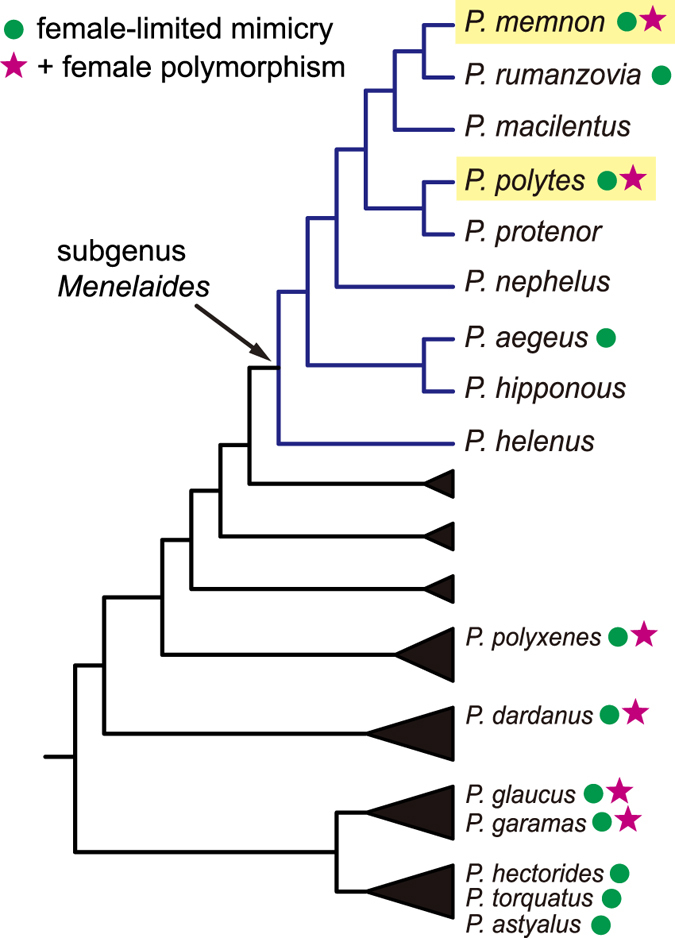
A sketch of the phylogeny and occurrence of female-limited mimicry polymorphism in the genus *Papilio* butterflies. The cladogram is drawn based on Zakharov *et al*.^16^. Species with circles show female-limited mimicry, and those with stars additionally show polymorphism in females^28^.

**Figure 3:**
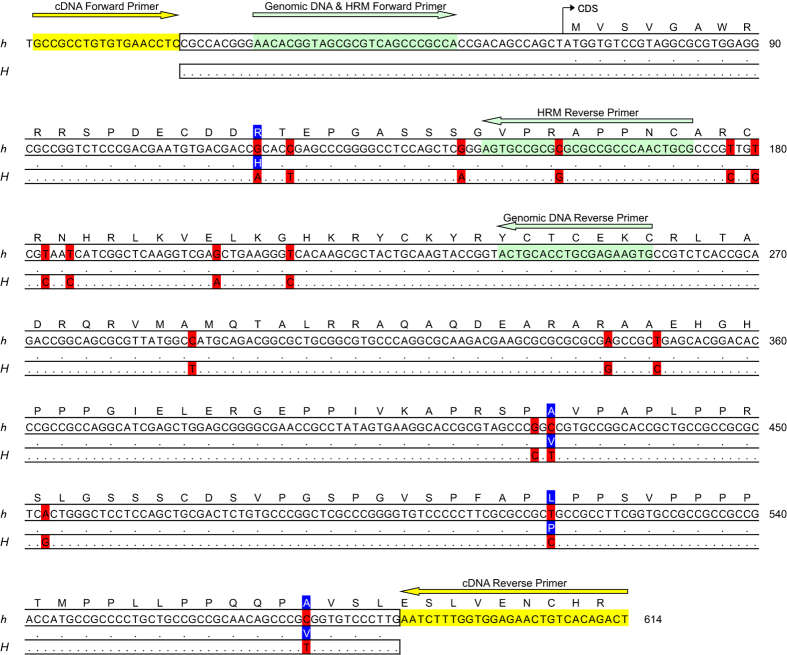
cDNA and amino acid sequences of the *Papilio memnon doublesex (dsx*) *H* and *h* alleles. The amino acid sequences are shown above the cDNA sequences. Dots indicate sequence identities in the *dsx H* and *h* alleles. Single-nucleotide polymorphisms are highlighted in red, and amino acid substitutions are highlighted in blue. The primers used are indicated above the sequences.

**Figure 4:**
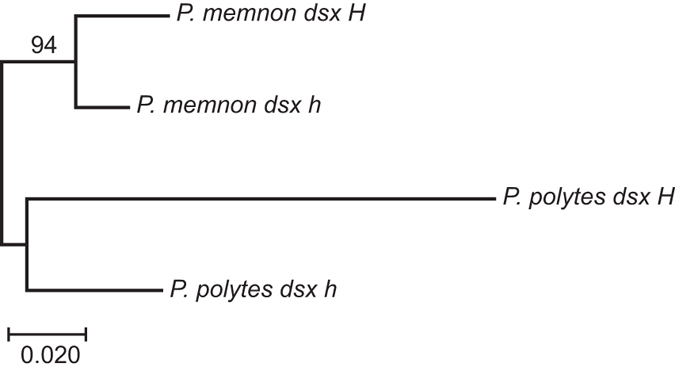
Maximum-likelihood tree for the relationship among *dsx H* and *h* alleles in *Papilio memnon* and *P. polytes*. The numeral above the branch is bootstrap percentage.

We found that the mimetic and non-mimetic forms of *P. memnon* females corresponded exactly with genotypes defined by two distinct allelic sequences in the *dsx* gene, suggesting that the genetic basis of female-limited Batesian mimicry in *P. memnon* is similar to that in *P. polytes*. It is not surprising that the same gene (i.e. *dsx*) controls mimicry polymorphism in the two closely related species. However, these two *Papilio* species are not sister species (Fig. 2), and the allelic *dsx* sequences of *P. memnon* differ considerably from those of *P. polytes*. Interestingly, all of the non-synonymous substitutions between the mimetic and non-mimetic alleles in *P. memnon* differed from those in *P. polytes*. The phylogenetic tree of the four *dsx* alleles (Fig. 4) revealed that the mimetic (*H*) and non-mimetic (*h*) alleles of *P. memnon* diverged independently from those of *P. polytes*. Thus, different mutation-selection processes in the *dsx* gene region may have resulted in the same pattern of female-limited mimicry polymorphism in these species. In the phylogeny of *Papilio*, the clade containing *P. memnon* and *P. polytes* and three other species includes two species without any sexual differences in terms of mimicry (Fig. 2). Therefore, even if the mimicry polymorphism evolved from their common ancestor, the responsible locus (including *dsx*) may have been subject to considerable evolutionary changes among the derived species. Furthermore, in *P. dardanus*, which is distantly related to *P. polytes* and *P. memnon*, the female-limited mimicry polymorphism has been found to be controlled by the transcription factor gene *engrailed*^13^. Female-limited mimicry evolved repeatedly in the genus *Papilio*, which originated approximately 55–65 million years ago^16^, and different mechanisms of controlling female-limited Batesian mimicry polymorphism may have evolved in different lineages. Further genomic studies are needed to reveal the origin of this female-limited Batesian mimicry polymorphism and its relationship with *dsx* in *Papilio* butterflies.

Batesian mimicry polymorphism is thought to be maintained by negative frequency-dependent selection^6^,^17^,^18^,^19^. However, few studies of female-limited Batesian mimicry polymorphism have attempted to estimate the frequencies of model, mimetic and non-mimetic forms^6^. To the best of our knowledge, no study exists on temporal or spatial variations in the frequencies of mimetic/non-mimetic alleles in natural populations. Our genotyping method using HRM analysis allows quick and precise estimation of allele frequencies in natural populations of *P. memnon* and facilitates study of the adaptive dynamics of the mimicry polymorphism. A particular merit of our approach is that allele frequencies can be estimated in male populations. Males are collected more frequently than are females, and more importantly, it is assumed that they are not subjected to differential predation mortality with respect to the mimicry locus genotype. Indeed, in this study, far fewer females than males were collected, both in Okinawa and Taiwan (Table 1).

In Taiwan, where the females are polymorphic, the mimetic allele (*H*) frequency in males was 45% on average and did not differ significantly among localities. In addition, the genotype frequencies at different sites did not depart from Hardy-Weinberg equilibrium, suggesting that mating was random in terms of mimetic alleles in the previous generation, and that the mimetic genotype did not affect juvenile survival. Only the non-mimetic allele was present in Okinawa, although the *P. memnon* population of Japan may have originated from Taiwan or another Southeast Asian region where both forms occur, and unpalatable papilionids *Pachliopta* and *Atrophaneura* occur in Okinawa. The absence of the mimetic allele in Okinawa may be attributed to selection against the mimetic allele, a founder effect, or genetic drift. The ecological and evolutionary factors responsible for maintenance or loss of female-limited Batesian mimicry polymorphism in *P. memnon* must be examined in both the field and laboratory, and our method of discriminating the mimicry genotypes will be useful in such studies.

## Methods

### Sequencing *dsx* mRNA

Two mimetic females of *P. memnon* were collected at Hualien in eastern Taiwan (23°59′N, 121°32′E). A laboratory colony developed from eggs laid by these females was reared successively (at 25 °C under a 14:10 L:D photoperiod) over three generations. The mimetic phenotype (genotype *HH* or *Hh*) is dominant^11^. One F_2_ mimetic female (expected genotype *Hh*) was hand-paired with one F_2_ male (expected genotype *hh*). Of their offspring (F_3_), 12 male and 7 female pupae were fixed in RNAlater (Qiagen) on the seventh day of pupation and stored at −30 °C prior to RNA extraction. Total RNA was extracted from developing wing discs of these pupae using the RNeasy Mini Kit (Qiagen), following the manufacturer’s protocol. Two total RNA samples from one male and one female pupa were sent to Hokkaido System Science Co. Ltd., Sapporo, Japan, for library construction using the Illumina TruSeq method (paired-end, 101 cycles) and for sequencing using the Illumina Hiseq 2000. All raw reads from the two individuals were assembled *de novo* using Trinity version_r20140717^20^ running the default settings. *Dsx* sequences were searched among the contigs using BLAST + 2.2.30^21^ using the F1 isoform sequences of the *dsx H* and *h* alleles of *P. polytes*^14^ as query sequences.

After obtaining *dsx* RNA contigs from *P. memnon*, the *dsx* RNAs of 11 F_3_ males and 6 F_3_ females (expected genotypes, *Hh* or *hh*) were sequenced to identify allele-specific sequences of *dsx H* and *h*. Extracted total RNAs were reverse-transcribed into single-stranded cDNAs using a ReverTra Ace^®^ qPCR RT Kit (Toyobo, Osaka, Japan). Based on the *dsx* mRNA contigs, we designed primers (forward, PmDsxf_F2: 5′-GCCGCCTGTGTGAACCTC-3′; reverse, PmDsxf_R8: 5′-AGTCTGTGACAGTTCTCCACCAAAGATT-3′) amplifying a 567-bp region of *dsx*. PCR amplification was performed in 8-μL volumes containing 5.72 μL ultrapure water, 0.64 μL 2.5 mM dNTP mix, 0.80 μL Ex-Taq buffer, 0.15 μL each primer (10 μM), 0.04 μL Ex-Taq DNA polymerase (Takara, Shiga, Japan) and 0.5 μL cDNA template. The PCR settings were (1) 94 °C for 3 min, (2) 30 cycles of 98 °C for 10 s and 68 °C for 60 s and (3) 72 °C for 7 min. The targeted region was GC-rich (70.6–71.0%), and the denaturation temperature in step (2) was thus set to a higher than normal temperature. The PCR products were sequenced directly on an ABI 3130xl sequencer (Applied Biosystems, Foster City, CA, USA) using the above primers and a BigDye Terminator Cycle Sequencing FS Ready Reaction Kit version 3.1 (Applied Biosystems). The nucleotide sequences were aligned using MEGA version 6.06^22^. Heterozygous sites within each individual were manually identified using 4peaks software^23^. The phylogenetic relationship among the 567-bp sequences of *dsx H* and *h* alleles in *P. memnon* and *P. polytes* was analysed by the maximum-likelihood method using MEGA version 6.06^22^. The substitution model used was GTR + G (general-time-reversible model with gamma distribution for rate heterogeneity); a bootstrap analysis with 1000 replications was performed.

### Collection of wild butterflies and sequencing

In total 134 *P. memnon* individuals were collected from nine localities in Taiwan and three localities on the main island of Okinawa in June and July 2013 (Table 1). Both mimetic and non-mimetic females occur in Taiwan, but only non-mimetic females are found in Okinawa. The whole bodies of the collected butterflies were fixed in absolute ethanol after recording the sex and, if applicable, wing type. Total genomic DNA was extracted using a Genomic DNA Purification Kit (Promega, Madison, WI, USA). To genotype the *dsx* locus, we designed primers for PCR-amplification of a 185-bp region of exon 1 including a part of the open reading frame: forward (alias, PmDsx_Hh_F9), 5′-AACACGGTAGCGCGTCAGCCCGCCA-3′; reverse (alias, PmDsxf_R2), 5′-CACTTCTCGCAGGTGCAGT-3′. The settings for PCR using Ex-Taq were (1) 94 °C for 3 min (2) 30 cycles of 98 °C for 10 s, 66 °C for 30 s and 72 °C for 30 s and (3) 72 °C for 7 min. The PCR products were sequenced directly (using the same primers) on the ABI3130xl sequencer.

### High-resolution melting analysis

HRM analysis using a real-time PCR platform is a rapid, economical, and sensitive genotyping tool for the study of natural populations^24^,^25^. For HRM analysis, we used the forward primer PmDsx_Hh_F9 described above and a newly designed reverse primer (alias: Pmemnon_HRM_R1), 5′-CGCAGTTGGGCGGCGCSCGCGGCACT-3′, based on the consensus sequences of the *dsx H* and *h* loci. These primers were used to amplify a 93-bp portion of exon 1. cDNAs from the 19 laboratory-reared butterflies and genomic DNA from the 156 field-collected butterflies were subjected to pre-amplification and HRM analysis using a LightCycler^®^ 96 System (Roche) with a LightCycler^®^ 480 High Resolution Melting Master (Roche) and the saturating dye ResoLight. Pre-amplification was performed in 10-μL volumes containing 0.3 μL ultrapure water, 5.0 μL 2 × master mix, 0.4 μL each primer (10 μM), 1.4 μL 25 mM MgCl_2_ and 2.5 μL template DNA. The PCR settings were (1) 95 °C for 10 min, (2) 45 cycles of 95 °C for 10 s, 65 °C for 15 s and 72 °C for 15 s, (3) 95 °C for 1 min and (4) 40 °C for 1 min. HRM analysis was performed from 65 °C to 97 °C, increasing at 0.03 °C/s with 25 readings per degree increase. LightCycler^®^ 96 SW 1.1 software was used to conduct the data analysis. The sensitivities of the delta *T*_*m*_ values and shape discriminations were adjusted manually to enable reliable genotyping.

The proportions of *dsx* genotypes at each locality in Taiwan were evaluated in terms of Hardy-Weinberg equilibrium using the “HardyWeinberg” package^26^ in R^27^. The geographical variations among *dsx* allele frequencies in Taiwan were analysed using Fisher’s exact probability test.

## Additional Information

**How to cite this article**: Komata, S. *et al*. Identification of *doublesex* alleles associated with the female-limited Batesian mimicry polymorphism in *Papilio memnon. Sci. Rep.* **6**, 34782; doi: 10.1038/srep34782 (2016).

## Supplementary Material

Supplementary Information

## Figures and Tables

**Figure 1:**
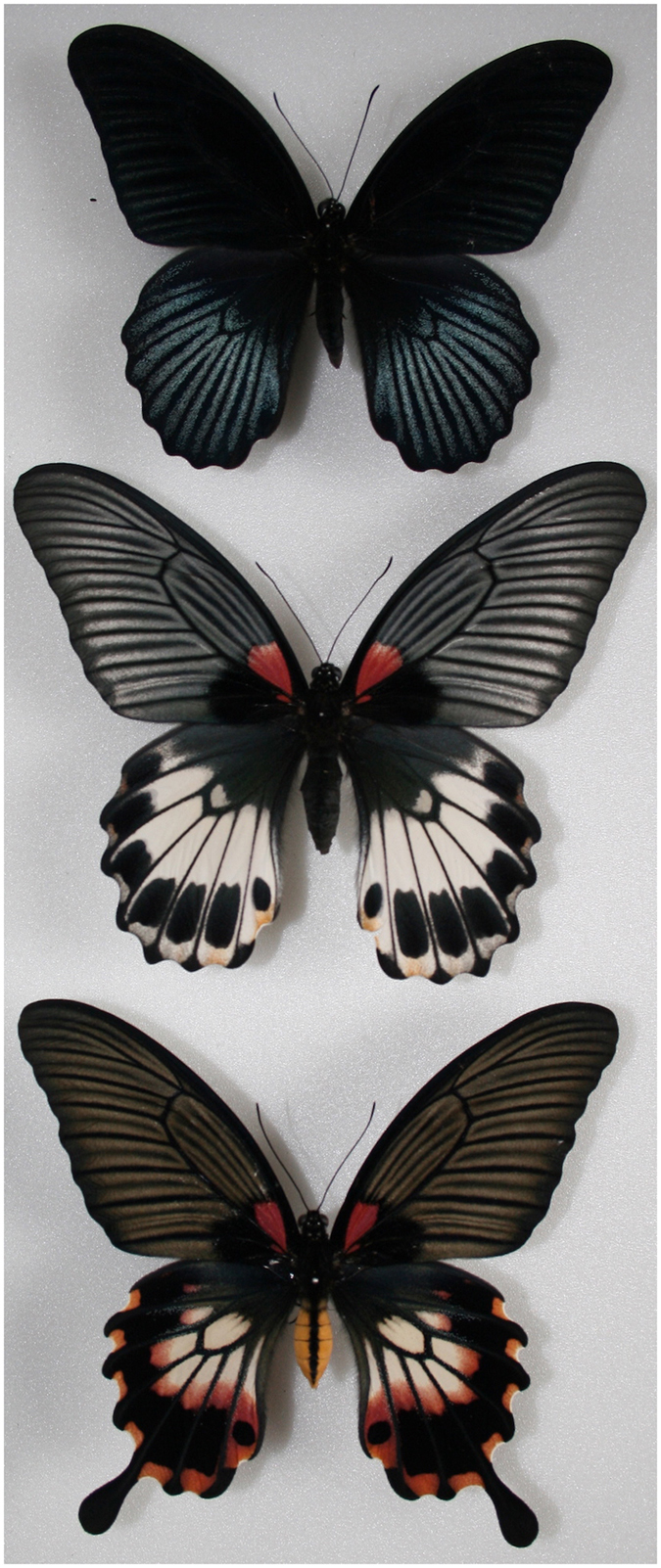
A male (top), non-mimetic female (middle) and mimetic female (bottom) of *Papilio memnon*.

**Table 1:** The number of samples used in this study and the *doublesex* allele types in *Papilio memnon* individuals collected in the wild, as determined by direct sequencing.

Locality	Male					Female				
	*n*	*HH*	*Hh*	*hh*	*H* freq.	*n (n*_m_, *n*_n_)†	*HH*	*Hh*	*hh*	*H* freq.
Okinawa										
Yona	25	0	0	25	0.00	1 (0, 1)	0	0	1	0.00
Uruma	3	0	0	3	0.00	1 (0, 1)	0	0	1	0.00
Naha	2	0	0	2	0.00	0	—	—	—	
Taiwan										
Hsinchu	11	3	5	3	0.50	2 (2, 0)	0	2	0	0.50
Miaoli	8	1	6	1	0.50	1 (0, 1)	0	0	1	0.00
Yilan	11	3	4	4	0.45	1 (1, 0)	0	1	0	0.50
Taichung	4	2	0	2	0.50	1 (1, 0)	0	1	0	0.50
Jian, Hualien	6	0	5	1	0.42	6 (4, 2)	0	4	2	0.33
Ruisui, Hualien‡	11	1 (2)	5 (4)	5 (5)	0.36	1 (1, 0)	1	0	0	1.00
Jiayi	10	4	3	3	0.55	5 (3, 2)	1	2	2	0.40
Gaoxiong	9	1	5	3	0.39	1 (0, 1)	0	0	1	0.00
Pingtung	9	1	6	2	0.44	5 (3, 2)	0	3	2	0.30

^†^“*n*_m_” and “*n*_n_” are the numbers of mimetic and non-mimetic females, respectively.

^‡^The numbers in parentheses are the genotype outcomes by HRM analysis. The statistical analysis was performed using the HRM data for this locality, assuming that genotyping by HRM analysis was correct (because direct sequencing might have featured some incorrect base calls).
