# Supplementary material for: Identification of doublesex alleles associated with the female-limited Batesian mimicry polymorphism in Papilio memnon
Source: Sci Rep. 2016 Oct 6;6:34782. doi: 10.1038/srep34782 (PMC5052519; doi:10.1038/srep34782)
Supplement: Supplementary Information [file srep34782-s1.pdf]

Supplementary Information for:

Identification of *doublesex* alleles associated with female-limited Batesian mimicry polymorphism in *Papilio memnon*

Shinya Komata[1], Chung-Ping Lin[2], Takuro Iijima[3], Haruhiko Fujiwara[3] & Teiji Sota[1]

[1]Department of Zoology, Graduate School of Science, Kyoto University, Kyoto, Japan.

[2]Department of Life Science, National Taiwan Normal University, Taipei, Taiwan.

[3] Department of Integrated Biosciences, University of Tokyo, Kashiwa, Japan.

Table S1. Summary statistics for *Papilio memnon de novo* transcriptome assemblies generated from 101 bp paired-end Illumina HiSeq RNA-Seq data.

Samples	Male	Female
Raw Reads	40,220,084	40,726,412
Contigs	39,035	45,887
Mean length (bp)	979	960
Median (bp)	543	525
Longest Contig (bp)	13,415	22,633
N50 (bp)	1,693	1,685

Table S2. Results of BLAST search for the *doublesex* orthologs in the contigs of *P. memnon* transcriptomes.

Query[1]	BLAST hit: Contig ID	Length (bp)	Score (bits)[2]	E value[3]
P. polytes dsx H (F1 isoform)	*P. memnon* male			
	c20128_g1_i1	395	451	$9e^{-126}$
	c13591_g1_i2	1653	82.4	$1e^{-14}$
	P. memnon female			
	c1658_g1_i1	839	1103	0.0
	c17110_g5_i1	938	887	0.0
	c15384_g1_i2	1635	69.4	$1e^{-10}$
	c15384_g1_i1	927	69.4	$1e^{-10}$
P. polytes dsx h (F1 isoform)	*P. memnon* male			
	c20128_g1_i1	395	436	$2e^{-121}$
	c21881_g1_i1	454	217	$3e^{-55}$
	P. memnon female			
	c1658_g1_i1	839	1177	0.0
	c17110_g5_i1	938	1153	0.0
	c17110_g2_i1	840	217	$4e^{-55}$

[1] Deposited at PapilioBase (http://papilio.bio.titech.ac.jp); *dsx h*, Gene ID: PpolytesGene0000070; *dsx H*, Gene ID: PpolytesGene0000001.

[2] Bit score represents the degree of similarity between the query (*P. polytes*) and the reference (*P. memnon*) sequences; larger values indicate greater similarity.

[3] E-value represents the expected number of times to obtain a bit score larger than the observed value by chance from the current dataset. A smaller E-value (with larger bit score) indicates greater similarity between the sequences. "0.0" indicates a very small value (close to zero).

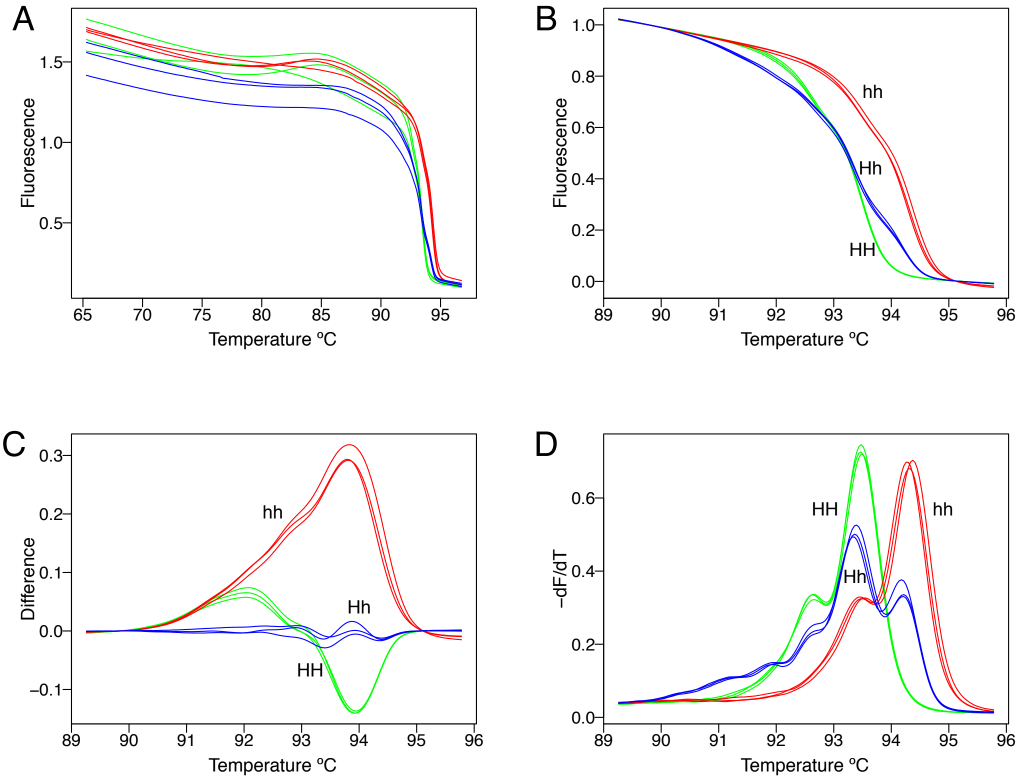

Figure S1. Identification of the *doublesex* allele types in *Papilio memnon* using high-resolution melting analysis. (A) Raw melting curves. (B) Normalized melting curves. (C) Difference plots. (D) Normalized melting peaks. The profiles are those of three heterozygotes (blue), three homozygous-dominant individuals (green) and three homozygous recessive individuals (red).
